# Estimated Cases of Blindness and Visual Impairment from Neovascular Age-Related Macular Degeneration Avoided in Australia by Ranibizumab Treatment

**DOI:** 10.1371/journal.pone.0101072

**Published:** 2014-06-30

**Authors:** Paul Mitchell, Neil Bressler, Quan V. Doan, Chantal Dolan, Alberto Ferreira, Aaron Osborne, Elena Rochtchina, Mark Danese, Shoshana Colman, Tien Y. Wong

**Affiliations:** 1 Department of Ophthalmology and Westmead Millennium Institute, University of Sydney, Westmead, New South Wales, Australia; 2 Wilmer Eye Institute, Johns Hopkins University, Baltimore, Maryland, United States of America; 3 Outcomes Insights, Inc., Westlake Village, California, United States of America; 4 CMD Consulting, Inc., Sandy, Utah, United States of America; 5 Novartis, Basel, Switzerland; 6 Genentech, Inc., South San Francisco, California, United States of America; 7 Singapore Eye Research Institute, National University of Singapore, Singapore, Singapore; 8 Centre for Eye Research Australia, University of Melbourne, Parkville, Victoria, Australia; Saitama Medical University, Japan

## Abstract

Intravitreal injections of anti-vascular endothelial growth factor agents, such as ranibizumab, have significantly improved the management of neovascular age-related macular degeneration. This study used patient-level simulation modelling to estimate the number of individuals in Australia who would have been likely to avoid legal blindness or visual impairment due to neovascular age-related macular degeneration over a 2-year period as a result of intravitreal ranibizumab injections. The modelling approach used existing data for the incidence of neovascular age-related macular degeneration in Australia and outcomes from ranibizumab trials. Blindness and visual impairment were defined as visual acuity in the better-seeing eye of worse than 6/60 or 6/12, respectively. In 2010, 14 634 individuals in Australia were estimated to develop neovascular age-related macular degeneration who would be eligible for ranibizumab therapy. Without treatment, 2246 individuals would become legally blind over 2 years. Monthly 0.5 mg intravitreal ranibizumab would reduce incident blindness by 72% (95% simulation interval, 70–74%). Ranibizumab given as needed would reduce incident blindness by 68% (64–71%). Without treatment, 4846 individuals would become visually impaired over 2 years; this proportion would be reduced by 37% (34–39%) with monthly intravitreal ranibizumab, and by 28% (23–33%) with ranibizumab given as needed. These data suggest that intravitreal injections of ranibizumab, given either monthly or as needed, can substantially lower the number of cases of blindness and visual impairment over 2 years after the diagnosis of neovascular age-related macular degeneration.

## Introduction

Neovascular age-related macular degeneration (AMD) is the leading cause of blindness in many developed countries, including Australia [1], [2]. Over the past 7 years, landmark clinical trials have shown that suppression of vascular endothelial growth factor (VEGF) with monthly or less frequent as-needed intravitreal injections of anti-VEGF agents prevented at least moderate visual acuity (VA) loss in nearly 95% of patients with neovascular AMD after 1 year and nearly 90% after 2 years, and at least moderate VA improvement has been noted in up to 40% of patients [3]-[5].

However, despite the clinical efficacy of this treatment and its widespread use in many countries, few studies have investigated the population-wide impact of anti-VEGF therapy on the incidence of blindness and visual impairment [6]. A Danish study recently showed that legal blindness attributable to AMD has halved since the introduction of anti-VEGF therapies and an Israeli study showed a reduction in overall blindness over time after anti-VEGF therapy [7], [8].

In the USA, a recent model estimated that the number of cases of legal blindness caused by neovascular AMD would reduce dramatically if monthly ranibizumab (Lucentis, Genentech, Inc., South San Francisco, CA, USA/Novartis AG, Basel, Switzerland) was used when indicated compared with no treatment [9]. Treatment was expected to reduce cases of legal blindness (defined in the USA as best-corrected visual acuity [BCVA] of 20/200 or worse in the better-seeing eye) by approximately 72% (95% confidence interval [CI], 70–74%) and visual impairment (defined as BCVA worse than 20/40 in both eyes) by approximately 37% (95% CI, 35–39%). These data suggested that the impact of neovascular AMD on legal blindness and visual impairment is reduced dramatically when monthly ranibizumab is available.

Additionally, a retrospective US study confirmed that the prevalence of legal blindness and visual impairment 2 years after the diagnosis of neovascular AMD has decreased substantially since the introduction of anti-VEGF therapy [10]. Some patients in this retrospective study received dosing as needed instead of monthly.

In Australia, AMD is the leading cause of blindness and visual impairment in individuals aged 65 years or older and has been estimated to cost the country over $5 billion per year (2010 figures) [11], [12]. The impact of ranibizumab therapy on the number of cases of legal blindness and visual impairment caused by neovascular AMD in Australia is unknown. Estimates from the recent model for the USA [9] are unlikely to be directly applicable to Australia due to potential differences in patient characteristics, incidence of neovascular AMD and treatment behaviours. In particular, the US model only considered patients receiving monthly ranibizumab treatment, which is only relevant to a subset of patients with neovascular AMD worldwide. In most other countries, including Australia, patients treated with ranibizumab for visual impairment due to neovascular AMD typically receive therapy on an as-needed basis. Thus, the aim of the present study was to estimate the proportion of cases of legal blindness and visual impairment due to neovascular AMD in Australia that were avoided by treatment with ranibizumab given monthly or as needed over 2 years. A model was constructed assuming that all eligible patients would receive treatment.

## Materials and Methods

### Subjects

**Figure 1:**
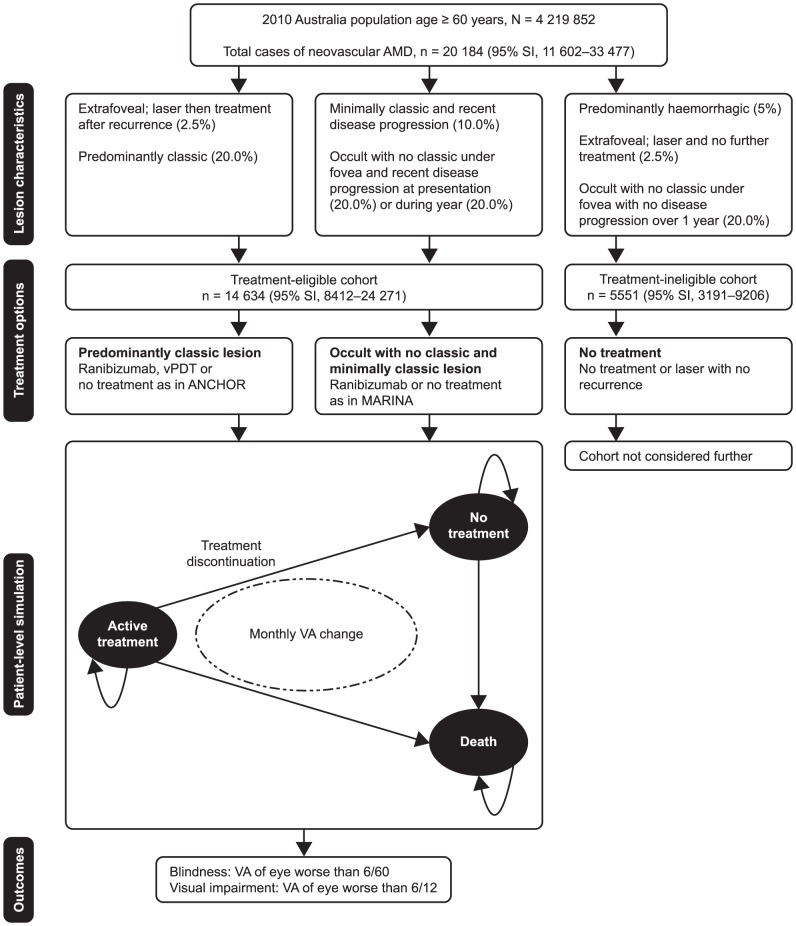
Model schematic. Incident cases of neovascular age-related macular degeneration (AMD) were derived by multiplying the number of individuals in each age group and gender by the respective incidences in the Blue Mountains Eye Study. Incident cases of neovascular AMD from 1 year were in the model for 2 years. Among individuals with AMD in one eye, 33% were estimated to have AMD in the fellow eye at baseline [3], [5], [14]. SI: simulation interval; VA: visual acuity; vPDT: photodynamic therapy with verteporfin.

The analysis was based on all Australians aged 60 years or over in 2010 (Table 1). Incident cases of neovascular AMD were derived from the estimated 10-year cumulative incidence of AMD in the Blue Mountains Eye Study (BMES) [13], extrapolated to the Australian population in 2010, and assuming that events occurred evenly over the observation period. Among individuals with neovascular AMD, it was assumed that 33% had existing neovascular AMD in the fellow eye at baseline using information from the Age-Related Eye Disease Study (AREDS), and ANCHOR and MARINA phase 3 ranibizumab trials [3], [5], [14]. Base-case distribution of lesion types was based on the population used in the recent US model [9]; 5% were predominantly haemorrhagic, 5% extrafoveal, 10% minimally classic, 20% predominantly classic and 60% occult. Patients were classified according to lesion type into three cohorts, which determined their eligibility for treatment (Figure 1). The ‘PC lesion’ cohort had predominantly classic lesions on fluorescein angiography; the ‘OC/MC lesion’ cohort had occult with no classic or minimally classic lesions; the ‘treatment-ineligible’ cohort had lesions that were considered, by the authors, as unlikely to receive ranibizumab treatment and this cohort was not included in the model.

**Table 1:** The Australian population aged 60 years or over in June 2010; distribution by age and gender.

Australian total population	60–69 years	70–79 years	≥ 80 years	Total
Male	1 053 685	599 729	326 947	**1 980 361**
Female	1 067 032	662 006	510 453	**2 239 491**
**Total**	**2 120 717**	**1 261 735**	**837 400**	**4 219 852**

Australian Bureau of Statistics (2011) 3101.0- Australian Demographic Statistics, Dec 2010. (Accessed September 2013 from http://www.abs.gov.au/AUSSTATS/abs@.nsf/mediareleasesbyCatalogue/251ECE081EC4B2EECA2579190013DCED).

### Model structure

The 2-year rates of blindness and visual impairment were estimated using a patient-level simulation developed in TreeAge Pro 2009 Suite (TreeAge Software, Inc., Williamstown, MA, USA) that included three primary health states: ‘active treatment’, ‘no treatment’ and ‘death’ (Figure 1). Each patient began the model on a specific treatment and remained on active treatment until discontinuation or death. The model accounted for VA changes in each eye, treatment discontinuation, risk of AMD in the fellow eye and mortality risk over each monthly interval for 2 years. Separate simulations were run for the PC lesion cohort and the OC/MC lesion cohort to estimate the 2-year rates of outcomes. These rates were then applied to the size of each cohort to determine the magnitude of the outcomes at the population level using @Risk for Excel (version 5.5.1; Palisade Corporation, Ithaca, NY, USA). Change over 2 years in patients with incident neovascular AMD in year 1 was simulated in the model. Model parameters are specified in Table 2.

**Table 2:** Specification of the model parameters.

Model parameter		Value	Data source
Mortality		Overall death rate: 5.63/1000; age- and gender-specific rates used	Australian Bureau of Statistics[16], [17]
Patients with health insurance/access problems		All residents of Australia are covered under Medicare plan and ranibizumab is fully covered for subfoveal neovascular AMD	Australian Health Service^a^
1-year incidence (SE) of neovascular AMD, women by age, years	< 60	0	BMES[13]
	60–69	0.0027 (0.0008)	
	70–79	0.0064 (0.0016)	
	≥ 80	0.0155 (0.0093)	
1-year incidence (SE) of neovascular AMD, men by age, years	< 60	0	BMES[13]
	60–69	0.0011 (0.0006)	
	70–79	0.0023 (0.0010)	
	≥ 80	0.0083 (0.0080)	
Patients with neovascular AMD in the fellow eye at baseline, %		33	Bressler *et al.* 2003[14]
Probability of developing neovascular AMD in the fellow eye, per month		0.0071	AREDS report number 8^b^
Baseline BCVA, LogMAR letter score, mean (SD) for the PC lesion cohort	Treated eye	46.5 (13.1)	ANCHOR trial data [3]; sampled from empirical trial data distribution
	Fellow eye without neovascular AMD at baseline	77.4 (13.7)	
	Fellow eye with neovascular AMD at baseline	34.5 (26.1)	
Baseline BCVA, LogMAR letter score, mean (SD) for the OC/MC lesion cohort	Treated eye	53.5 (13.2)	MARINA trial data;[5] sampled from empirical trial data distribution
	Fellow eye without neovascular AMD at baseline	76.1 (14.7)	
	Fellow eye with neovascular AMD at baseline	38.6 (26.2)	
Distribution of lesion subtypes, %	No treatment	27.5	Assumptions established in Bressler *et al.* 2011[9]
	PC lesion cohort	22.5	
	OC/MC lesion cohort	50.0	
Change in BCVA at 24 months		From empirical distributions	ANCHOR[3] and MARINA[5] trial data
Difference between monthly versus as-needed ranibizumab dosing in BCVA change at 24 months, letters (95% CI)		−2.1 (−5.2–1.0)	CATT study data[4]
Treatment discontinuation, monthly probability	Ranibizumab (PC lesions)	0.00178	ANCHOR (unpublished data, 2009)[9]
	Ranibizumab (OC/MC lesions)	0.00173	MARINA (unpublished data, 2006)[9]
	Photodynamic therapy	0.00407	ANCHOR (unpublished data, 2009)[9]
Patients, by BCVA letter score, after 2 years without treatment in PC lesion cohort, % (SD)	≤ 38 (worse than 6/60) in incident eye	67 (5.16)	TAP report number 3^c^ (predominantly classic CNV)^d^, SD reported in Bressler *et al*. 2011[9]
	≤ 38 (worse than 6/60) in better-seeing eye	22.3 (0.05)	TAP report number 3^d^ and ANCHOR[3]
	≤ 68 (worse than 6/12) in incident eye	97.0 (1.86)	Assumption from Bressler *et al*. 2011[9]
	≤ 68 (worse than 6/12) in both eyes	52.6 (0.10)	Estimated based on TAP report number 3^c^^,^ d
BCVA change per month after discontinuation from active treatment, %		1.6	Based on 2-year sham-treatment results in MARINA (−14.9 letters in 24 months)[5]

a Pharmaceutical Benefits Scheme. Ranibizumab. (Accessed September 2013 from www.pbs.gov.au). 2011.

b A randomized, placebo-controlled, clinical trial of high-dose supplementation with vitamins C and E, beta carotene, and zinc for age-related macular degeneration and vision loss: AREDS report no. 8. *Arch Ophthalmol* 2001; 119: 1417–1436.

c The mean baseline visual acuity of patients in TAP report number 3 is a 50-letter score.

d Bressler NM, Arnold J, Benchaboune M, Blumenkranz MS, Fish GE, Gragoudas ES *et al.* (2002) Verteporfin therapy of subfoveal choroidal neovascularization in patients with age-related macular degeneration: additional information regarding baseline lesion composition's impact on vision outcomes-TAP report No. 3. *Arch Ophthalmol* 120: 1443–1454.

AMD: age-related macular degeneration; BCVA: best-corrected visual acuity; CI: confidence interval; CNV: choroidal neovascularization; OC/MC lesion: occult with no classic lesions or minimally classic lesions; PC lesion: predominantly classic lesions; SD: standard deviation; SE: standard error.

### Treatments

The treatment alternatives were ranibizumab 0.5 mg, given monthly (specified as every 30 ± 7 days) [3], [5], ranibizumab dosed as needed (i.e. according to signs of AMD as detected on 4-weekly optical coherence tomography [OCT], as used in the Comparison of AMD Treatment Trials [CATT] study) [4], and photodynamic therapy (PDT) with verteporfin (vPDT) or no treatment if vPDT was not indicated. Across these scenarios, all eligible patients in the model received only the specified treatment. The PC lesion cohort received treatment similar to patients in the ANCHOR trial (ranibizumab, vPDT or no treatment) and the OC/MC lesion cohort received treatment as received by patients in the MARINA trial (ranibizumab or no treatment).

### Baseline visual acuity and visual acuity change

The baseline VA for the PC lesion and OC/MC lesion cohorts was based on BCVA distributions in the treated and fellow eyes for patients in ANCHOR and MARINA, respectively [3], [5]. Because the results from a subgroup analysis of ANCHOR suggested that the extent of VA change is conditional on baseline VA, the Early Treatment Diabetic Retinopathy Study [ETDRS] chart letter score change over 2 years was sampled from the same patients selected at baseline to preserve the relationship between baseline VA and VA change [15]. For monthly ranibizumab treatment, the VA change from each study and each treatment was applied to the corresponding neovascular AMD lesion subtype and treatment group in the model. For ranibizumab dosed as needed, it was assumed that the gain in VA letter score achieved at 24 months was 2.1 (95% CI, −1.0–5.2) less than that achieved with monthly dosing, based on 2-year data from the CATT study [4]. This adjustment was applied to the patient-level ANCHOR and MARINA data. The model also accounted for the risk of treatment discontinuation each month using discontinuation rates from the ANCHOR and MARINA trials. While the patient was not receiving treatment, VA change was assumed to decline by 1.6% per month based on the 2-year sham-treatment results in MARINA (a loss of 14.9 letters over 24 months) [5]. Patients could not return to active treatment after discontinuation. The VA letter scores in each eye were tracked for each month up to month 24 or the time of death, whichever occurred first. A monthly risk of death was applied using Australian age- and gender-specific mortality data [16], [17].

### Model outcomes

The key outputs from the model were the number of cases of legal blindness, defined as a VA score worse than 6/60 (approximated as a letter score of 38) in the better-seeing eye, and the number of cases of vision impairment, defined as a VA score of worse than 6/12 (approximated as a letter score of 68) in the better-seeing eye, over 2 years, including those patients already classified as having legal blindness [18]. For these outcomes, ranibizumab was compared against no treatment, because PDT is now rarely used in Australia.

### Sensitivity analyses

One-way sensitivity analyses were conducted on the proportions of neovascular AMD lesion types to assess the impact of these on blindness and visual impairment. Probabilistic sensitivity analysis was undertaken to account for various sources of patient variability and parameter uncertainty. Whenever possible, the distribution of patient-level characteristics was informed by the patient-level variability from trial data (e.g. baseline VA of each eye, VA change at 24 months in each eye). Parameter uncertainty was characterized as either a normal or gamma distribution. Patient-level variability was sampled in the first level, while parameter uncertainty was sampled in the second level, of a two-dimensional Monte Carlo simulation. To achieve stable rates, 300 averages of 10 000 iterations were sampled. Most of the key inputs into the model (Table 2) were evaluated. The confidence in the results is reported as an interval around the expected mean that captured 95% of all possible simulated values (95% simulation interval [SI]) for each outcome.

## Results

### Incidence of neovascular AMD

The model predicted that 20 184 (95% SI, 11 602–33 477) people would have developed neovascular AMD in Australia in 2010. Of these, 33% (6728) would have had pre-existing neovascular AMD in the fellow eye at the start of 2010.

### Patients ineligible to receive ranibizumab

As shown in Figure 1, approximately 27.5% of incident cases of neovascular AMD (n  =  5551) would have lesion types that would not be considered eligible for ranibizumab treatment. Assuming that all of the predominantly haemorrhagic cases progressed to a VA of worse than 6/60, and 33% of the extrafoveal and occult with no classic cases for which treatment was not judged to be indicated developed similar disease in the fellow eye, 2553 individuals would become legally blind over the following 2 years.

### Patients eligible to receive ranibizumab

If none of the patients eligible to receive ranibizumab who were included in the model received treatment (n  =  14 634; 95% SI, 8412–24 271), 2246 (95% SI, 1300–3695) patients would become legally blind over 2 years (Table 3). If they all received ranibizumab on a monthly basis, this number would drop to 624 patients (95% SI, 357–1031), a decrease of 72% (95% SI, 70–74%). If ranibizumab was dosed as needed, 724 patients (95% SI, 414–1211) would become blind, a decrease of 68% (95% SI, 64–71%). Treatment with PDT in those eligible to receive it would result in a 12% (95% SI, 10–15%) reduction in cases of blindness (n  =  1968; 95% SI, 1141–3220) compared with no treatment.

**Table 3:** Blindness and visual impairment outcomes in patients with neovascular age-related macular degeneration with and without monthly treatment with ranibizumab.

Scenario	Number of patients (% of total cohort of 14 634)	95% SI, n (%)	Relative risk reduction compared with no treatment, % (95% SI)
**Legal blindness (BCVA worse than 6/60 in better-seeing eye** a **)**			
No treatment	2246 (15)	1300–3695 (9–25)	–
Monthly ranibizumab	624 (4)	357–1031 (2–7)	72 (70–74)
Ranibizumab dosed as needed	724 (5)	414–1211 (3–8)	68 (64–71)
PDT scenario: PDT indicated and accessible; ranibizumab not accessible	1968 (13)	1141–3220 (8–22)	12 (10–15)
**Visual impairment (BCVA worse than 6/12 in better-seeing eye** b **)**			
No treatment	4846 (33)	2782–8027 (19–55)	–
Monthly ranibizumab	3072 (21)	1763–5114 (12–35)	37 (34–39)
Ranibizumab dosed as needed	3504 (24)	1990–5833 (14–40)	28 (23–33)
PDT scenario: PDT indicated and accessible; ranibizumab not accessible	4773 (33)	2750–7884 (19–54)	1 (−1–4)
**BCVA worse than 6/60 in the incident eye**			
No treatment	7865 (54)	4534–13 120 (31–90)	–
Monthly ranibizumab	2538 (17)	1463–4197 (10–29)	68 (66–70)
Ranibizumab dosed as needed	2791 (19)	1604–4625 (11–32)	65 (61–68)
PDT scenario: PDT indicated and accessible; ranibizumab not accessible	7635 (52)	4433–12 616 (30–86)	3 (−3–8)
**BCVA worse than 6/12 in the incident eye**			
No treatment	8676 (59)	4987–14 456 (34–99)	–
Monthly ranibizumab	5632 (38)	3237–9336 (22–64)	35 (33–36)
Ranibizumab dosed as needed	6125 (42)	3513–10 166 (24–69)	29 (26–33)
PDT scenario: PDT indicated and accessible; ranibizumab not accessible	8606 (59)	4943–14 271 (34–98)	1 (−2–2)

a Legal blindness was defined as a BCVA letter score worse than 6/60 (approximate ETDRS letter score ≤ 38) in the better-seeing eye.

b Visual impairment was defined as a BCVA letter score worse than 6/12 (approximate ETDRS letter score ≤ 68) in the better-seeing eye.

BCVA: best-corrected visual acuity; ETDRS: Early Treatment Diabetic Retinopathy Study; PDT: photodynamic therapy; SI: simulation interval.

As summarized in Table 3, substantial reductions in the risk of bilateral visual impairment (BCVA worse than 6/60 in the incident eye) were predicted for ranibizumab dosed monthly or as needed. Bilateral visual impairment was predicted to develop over the 2-year period in 4846 patients with no treatment (95% SI, 2782–8027). Risk reductions of 37% and 28%, compared with no treatment, would be achieved with monthly ranibizumab and ranibizumab dosed as needed, respectively (Table 3). The corresponding reduction with PDT would be 1%. A BCVA worse than 6/60 in the incident eye would occur in 7865 patients (95% SI, 4534–13 120) with no treatment over the 2-year period; treatment with monthly or as-needed ranibizumab would achieve risk reductions of 68% and 65%, respectively, compared with no treatment, and the risk reduction for PDT would be 3%. A BCVA worse than 6/12 in the incident eye would occur in 8676 (95% SI, 4987–14 456) patients with no treatment, with risk reductions of 35%, 29% and 1% achieved with monthly ranibizumab, ranibizumab dosed as needed and PDT, respectively.

### Sensitivity analyses

**Figure 2:**
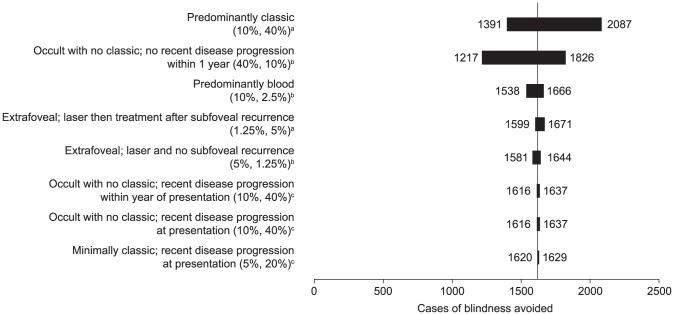
Sensitivity analyses. The impact of neovascular age-related macular degeneration lesion type on the cases of blindness (best-corrected visual acuity in better-seeing eye worse than 6/60) avoided using a monthly ranibizumab scenario compared with a no-treatment scenario. In the base analysis, 1622 cases of legal blindness were avoided with monthly ranibizumab, as indicated by the vertical line. ^a^Eligible for PDT and ranibizumab. ^b^Ineligible for any treatment. ^c^Eligible for ranibizumab, but not for PDT. PDT: photodynamic therapy.

**Figure 3:**
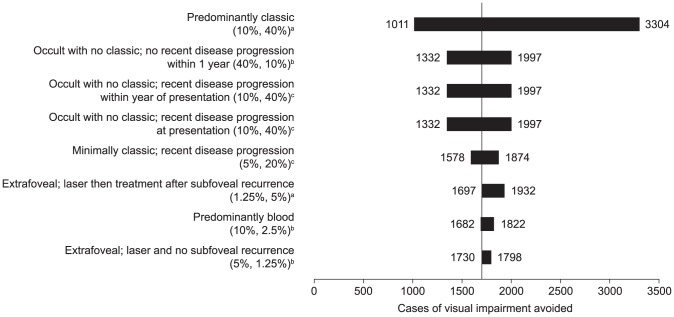
Sensitivity analyses. The impact of neovascular age-related macular degeneration lesion type on the cases of visual impairment (best-corrected visual acuity in better-seeing eye worse than 6/12) avoided using a monthly ranibizumab scenario compared with a no-treatment scenario. In the base analysis, 1774 cases of visual impairment were avoided with monthly ranibizumab, as indicated by the vertical line. ^a^Eligible for PDT and ranibizumab. ^b^Ineligible for any treatment. ^c^Eligible for ranibizumab, but not for PDT. PDT: photodynamic therapy.

In the one-way sensitivity analyses, the proportions of patients with each neovascular AMD lesion type had the greatest impact on the cases of blindness and visual impairment avoided (Figure 2 and Figure 3, respectively). The SIs derived from the probabilistic sensitivity analyses showed moderate uncertainty around the estimates of legal blindness and visual impairment (Table 3).

## Discussion

This study estimated the number of cases of blindness and visual impairment caused by neovascular AMD that can be avoided in Australia through the use of intravitreal ranibizumab injections. This model builds and expands on previous work by Bressler *et al*. [9] by accounting for characteristics specific to the Australian population, including Australian incidence data for neovascular AMD, Australian definitions for legal blindness and visual impairment, and Australian AMD incidence characteristics from population-level data. We modelled VA outcomes using 2-year, phase 3 ranibizumab trial data, which allowed us to use patient-level profiles following monthly treatment [3], [5]. Results from the CATT study were used to estimate cases of blindness avoided with as-needed treatment [4]. The inclusion of as-needed therapy is a further important expansion of the previous model [9] because in many countries, including Australia, patients treated with ranibizumab for visual impairment due to neovascular AMD typically receive therapy on an as-needed basis. Furthermore, the inclusion of both monthly and as-needed regimens for the same population permits a comparison of predicted outcomes.

The model predicted that 20 184 people would develop AMD in Australia in 2010. Of these, about 5500 patients would not be eligible for ranibizumab treatment. Without treatment, 2246 of the remaining patients would become legally blind over a 2-year period. The results suggest that monthly ranibizumab would reduce the risk of legal blindness by 72% compared with no treatment, while the risk of visual impairment would be reduced by 37%. Dosing ranibizumab as needed with monthly monitoring provides a comparable reduction in the risk of blindness and visual impairment: 68% and 28%, respectively. Given the comparable VA outcomes between monthly and as-needed dosing regimens observed in the CATT study over 2 years [4], it is not surprising that there were only slightly fewer cases of blindness avoided with as-needed treatment than with monthly treatment, despite less frequent dosing. The extent to which these benefits can be extended beyond the 2-year period considered in this study is currently unknown.

The results of this modelling exercise confirm findings from a real-world database study in Denmark [7]. In that study, cases of blindness due to neovascular AMD were halved during the latter half of a 10-year period during which ranibizumab was introduced with a similar availability as in Australia. Since that study assessed registered blindness and might not have used high-contrast charts to evaluate vision, it is difficult to compare the results with those of our study; however, the Danish study suggests that benefits can be extended beyond the 2-year window that we considered. Although comparison with real-world data was not the aim of this study, future validation of our findings with real-world data on the impact of anti-VEGF therapy in the Australian population is warranted. To date, no studies have been conducted to evaluate real-world reductions in blindness or visual impairment due to neovascular AMD in Australia following the introduction of anti-VEGF therapies. However, several ongoing studies may provide suitable real-world data for future validation in the Australian population [19], [20] or for comparison with the UK [21].

Particularly in older populations, blindness and visual impairment have been shown to have substantial clinical, humanistic and economic impacts including increased risk of falls and fractures, reduced mobility and independence, earlier need for supportive care (e.g. entry into a nursing home), and increased risk of mortality [22]-[30]. Thus, our findings may be of value to healthcare policy experts, recognizing that our results are theoretical in nature and need to be considered in context along with factors such as patient preferences for therapy, treatment costs and healthcare resources. The information provided in this study may also be of value to health economists for incorporation in future cost-effectiveness models.

This study has some limitations that need to be considered. First, the incidence rates of neovascular AMD were based on the BMES and there was no allowance for national variability; however, this study is considered representative of the portion of the Australian population that is affected by neovascular AMD (i.e. the Caucasian population), and applies to the older population of Australia in this study [31]. Secondly, there is a lack of patient-level data to estimate results for as-needed dosing. Nevertheless, since the VA profile found in the CATT study after treatment was consistent across both groups (monthly and as-needed ranibizumab) [4], it was considered appropriate to also use the MARINA[5] and ANCHOR[3] profiles for as-needed dosing. Thirdly, there is limited evidence for the distribution of lesion types in patients in Australia. Since lesion type has an impact on avoidable blindness, according to the sensitivity analysis, it is important to know which types of lesions occur in the real world and how they respond to ranibizumab. Fourthly, the results might not be generalizable when the frequency of treatment is reduced below that used in the CATT study, or when monitoring of disease activity is limited to less than the monthly monitoring used in the CATT study. Also, further work is needed to understand the impact of ‘treat-and-extend’ regimens, as used in some countries, on the prevention of blindness.

To conclude, the results of this study suggest that, in Australia, ranibizumab given for neovascular AMD would reduce the number of cases of legal blindness over a 2-year period by 68% and 72%, and the number of cases of visual impairment by 28% and 37%, with as-needed and monthly treatment, respectively. These results are consistent with the known clinical benefits of anti-VEGF therapy for neovascular AMD, and extend the positive impacts of this treatment to the Australian population. In the future, neovascular AMD may no longer be the leading cause of blindness in older adults in Australia.
